# The role of rhizobial (NifV) and plant (FEN1) homocitrate synthases in *Aeschynomene*/photosynthetic *Bradyrhizobium* symbiosis

**DOI:** 10.1038/s41598-017-00559-0

**Published:** 2017-03-27

**Authors:** Nico Nouwen, Jean-François Arrighi, Fabienne Cartieaux, Clémence Chaintreuil, Djamel Gully, Christophe Klopp, Eric Giraud

**Affiliations:** 1 IRD, UMR LSTM, Montpellier, F-34398 France; 2 INRA, Plateforme GenoToul Bioinfo, Castanet-Tolosan, F-31326 France

## Abstract

In the most studied rhizobium-legume interactions, the host plant supplies the symbiont with homocitrate, an essential co-factor of the nitrogenase enzyme complex, via the expression of a nodule-specific homocitrate synthase FEN1. Photosynthetic bradyrhizobia interacting with Nod factor (NF) dependent and NF-independent *Aeschynomene* legumes are able to synthesize homocitrate themselves as they contain a *nifV* gene encoding a homocitrate synthase. Here, we show that in the model strain ORS285, *nifV* is required for free-living and symbiotic dinitrogen fixation with NF-independent *Aeschynomene* species. In contrast, in symbiosis with NF-dependent *Aeschynomene* species, the *nifV* requirement for efficient nitrogen fixation was found to be host plant dependent. Interestingly, orthologs of *FEN1* were found in both NF-dependent and NF-independent *Aeschynomene* species. However, a high nodule specific induction of *FEN1* expression was only observed in *A. afraspera*, a host plant in which *nifV* is not required for symbiotic dinitrogen fixation. These data indicate that efficient symbiotic nitrogen fixation in many of the tested *Aeschynomene* species requires rhizobial homocitrate synthesis. Considering that more than 10% of the fully sequenced rhizobium strains do contain a *nifV* gene, the *Aeschynomene*/photosynthetic *Bradyrhizobium* interaction is likely not the only rhizobium/legume symbiosis where rhizobial *nifV* expression is required.

## Introduction

Nitrogen is an essential element for all living organisms. On earth the major source of nitrogen is atmospheric dinitrogen, which is fixed by microorganism (diazotrophs) that are able to reduce dinitrogen to ammonium by a nitrogenase enzyme complex. Leguminous plants establish a nitrogen-fixing symbiotic interaction with soil bacteria commonly called rhizobia. This symbiosis is a major contributor to the global nitrogen cycle and enables the host legumes to grow without an exogenous nitrogen source. In general, rhizobia induce the formation of a new organ, the nodule, on the roots of their host plant. The plant cells in the nodule are colonized intracellularly by the rhizobia which differentiate into an endosymbiotic form, the bacteroids, able to reduce atmospheric dinitrogen to the benefit of the plant. In turn, the plant supplies the bacteroids with the required carbon sources.

For nitrogen fixation, rhizobia use a molybdenum (Mo)-nitrogenase (EC 1.18.2.1). The enzymatic complex is composed of two components: the Fe protein and the MoFe protein containing a P-cluster and iron-molybdenum cofactor (FeMo-co). In the well-studied free-living diazotroph, *Klebsiella pneumonia*, the formation of an active nitrogenase enzyme complex depends on a cluster of 20 (*nif*) genes (for review see: ref. 1). The structural genes for the Fe protein and α- and β-subunits of the MoFe protein are encoded by the *nifH*, *nifD* and *nifK* genes, respectively. However, the synthesis of these structural proteins is not sufficient to obtain an active nitrogenase complex. It requires additional *nif* genes which play a role in the biosynthesis and the assembly of the FeMo-cofactor, in electron transport and in nitrogenase regulation (see for review: ref. 2). In contrast to free-living diazotrophs, the number of *nif* genes in rhizobia is very variable (for review see: ref. 3). Remarkably, the majority of the rhizobia lack the *nifV* gene which encodes a homocitrate synthase that catalyzes the condensation of acetyl coenzyme A and 2-oxoglutarate. Homocitrate is a component of the FeMo-cofactor present in the catalytic center of dinitrogenase that is absolutely required for a proper functioning of the nitrogenase enzyme complex^4^ which raises the question how the rhizobia are able to fix nitrogen during symbiosis. Recently, it has been demonstrated that *Lotus japonicus* expresses a nodule specific homocitrate synthase (FEN1) that compensates for the absence of homocitrate synthase activity in the bacterial partner *Mesorhizobium loti* ^5^. *Azorhizobium caulinodans* ORS571 and photosynthetic *Bradyrhizobium* strains are examples of rhizobia that contain a *nifV* gene. However, as these rhizobia are capable to fix dinitrogen under free-living conditions^6, 7^, we can ask if the presence of the *nifV* gene in these rhizobia is related to this capacity or because their host plant are unable to supply homocitrate during symbiosis.

To investigate this question, we have constructed a *nifV* deletion mutant in the photosynthetic *Bradyrhizobium* strain ORS285. We preferentially selected this strain as it can establish a nitrogen symbiosis with two distinct groups of *Aeschynomene* species that are discriminated by their use of a Nod-Factor (NF) dependent or a NF-independent mechanism to establish a symbiotic interaction. We correlated the nodulation phenotypes with the presence and expression of *FEN1* orthologs in *A. afraspera* and *A. evenia*, representatives of the NF-dependent and NF-independent *Aeschynomene* groups, respectively.

## Results

### Deletion of *nifV* results in a reduced nitrogenase enzyme activity in free-living conditions

**Figure 1:**
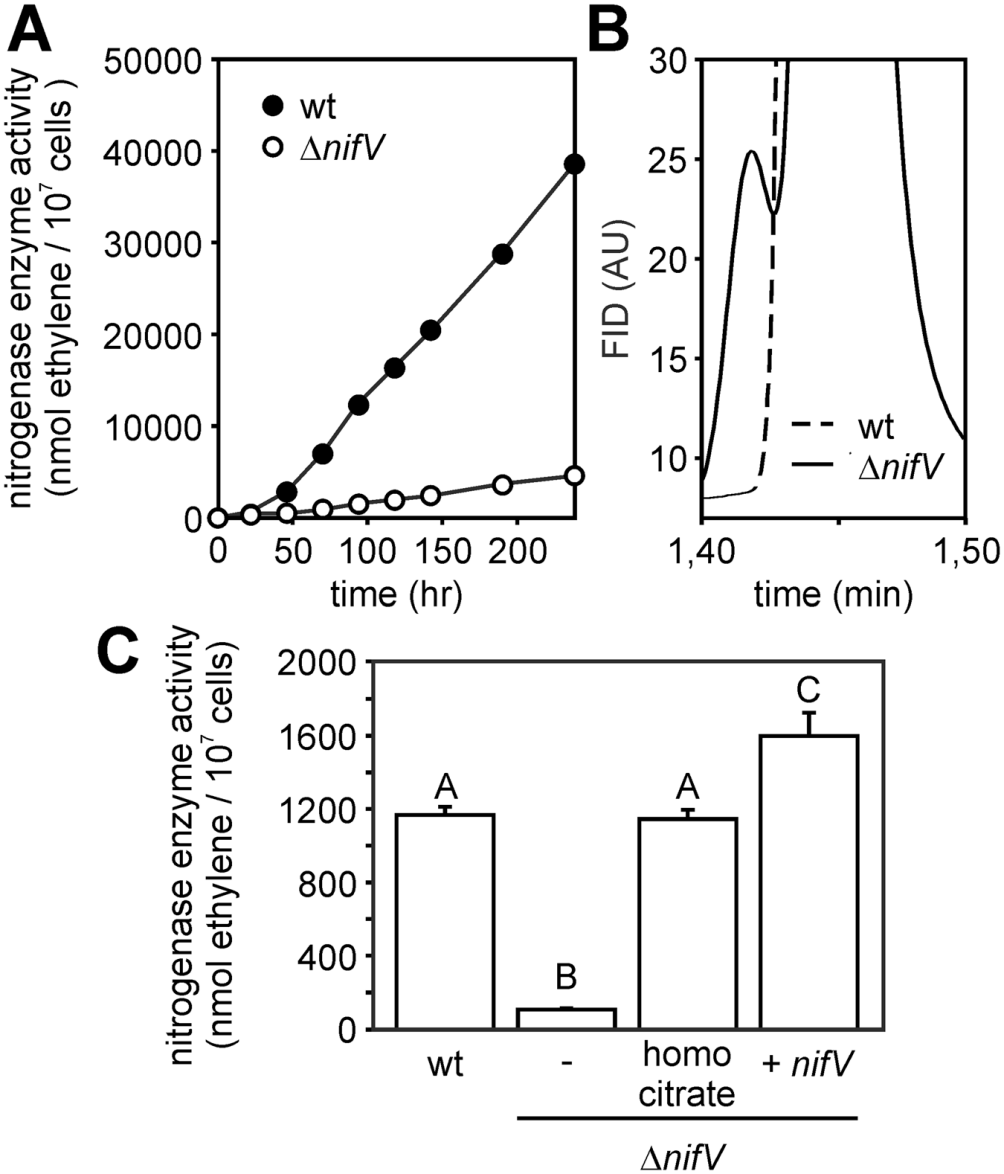
ORS285 Δ*nifV* mutant displays low *in vitro* acetylene reducing activity which is restored by homocitrate addition or re-introducing a complete *nifV* gene. (**A**) Ethylene production by ORS285 and ORS285 Δ*nifV* cultures grown in 150 ml vials containing BNM-B medium and 10% acetylene gas at different times post-inoculation. (**B**) Chromatogram of gas-samples taken from ORS285 (wt) and ORS285 Δ*nifV* cultures 8 days post-inoculation. WT: dashed line. Δ*nifV* mutant: solid line. (**C**) Ethylene production of ORS285 and derivatives grown for 7 days in vacuette® tubes containing BNM-B medium and 10% acetylene gas in the absence or presence of 10 mM homocitrate. The mean amount of produced ethylene per tube (n = 3) is indicated. Error bars represent standard errors of the mean. An ANOVA was performed among the different conditions, followed by Tukey’s post hoc analysis (P < 0.05). The different letters indicate groups that differ significantly.

Genomic analysis revealed the presence of one *nifV* gene in the ORS285 strain. This gene is localized in a chromosomic region that contains other *nif* genes (*nifH/Q/P/W*) and genes that have been shown to be necessary for an efficient nitrogen fixation (*fixABCX*, *mopBmodCD*) (Fig. S1). To analyze the importance of *nifV* in nitrogen fixation, we have constructed a *nifV* deletion mutant by double crossing over and analyzed its nitrogenase activity under free-living conditions using the acetylene reduction assay (ARA). Under growth conditions that induce nitrogenase genes, i.e. under low oxygen tension and absence of a combined source of nitrogen, the kinetics of ethylene formation by the Δ*nifV* mutant was drastically reduced as compared to the WT strain (Fig. 1A). Interestingly, analysis of the chromatograms revealed that gas samples from the Δ*nifV* mutant contained an additional volatile molecule eluting just before ethylene (Fig. 1B). A similar volatile molecule was observed in gaschromatograms from *nifV* mutants of other diazotrophs and identified as ethane^8, 9^. Therefore, the additional peak in the gaschromatogram we have indicated as “ethane” in the rest of the text. The addition of homocitrate to the growth medium or the re-introduction of a plasmid containing the *nifV* gene restores completely the nitrogenase activity of the Δ*nifV* mutant (Fig. 1C; Fig. S2A and B). This indicates that the observed effects in the Δ*nifV* mutant are solely due to the absence of the homocitrate synthase NifV and not to pleiotropic effects of the *nifV* deletion on the expression of downstream genes (*cysE*/*nifW*).

### Rhizobial homocitrate synthase is required for an efficient symbiotic interaction with NF-independent *Aeschynomene* species

**Figure 2:**
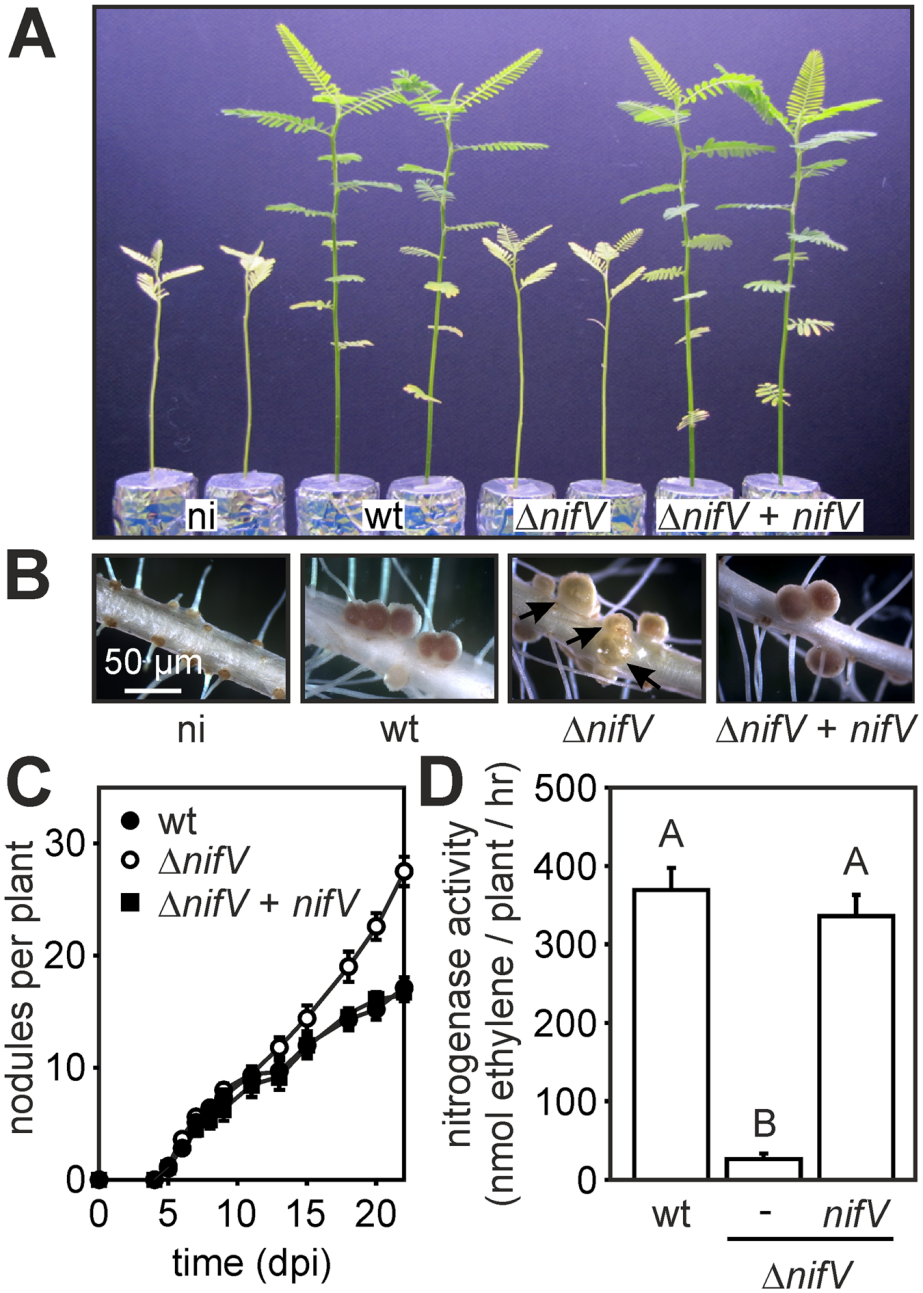
ORS285 *nifV* deletion affects the symbiotic interaction with *Aeschynomene evenia* (CIAT22838). (**A**) Comparison of the growth of *A. evenia* plants inoculated with ORS285, ORS285 Δ*nifV* and ORS285 Δ*nifV*+pMG105-*nifV*. Non-inoculated plants (ni) were used as control. (**B**) Mature nodules on *A. evenia* plants inoculated with different ORS285 derivatives. Note the presence of green colored nodules (indicated with a black arrow) on *A. evenia* plants inoculated with the ORS285 Δ*nifV* strain. (**C**) Nodulation kinetics of *Bradyrhizobium* ORS285 (wt), Δ*nifV* and Δ*nifV*+pMG105-*nifV* (Δ*nifV*+*nifV*) derivatives on *A. evenia* plants. The mean number of nodules per plant (n = 10) at various days post infection (dpi) is presented. Error bars represent standard errors of the mean. (**D**) Acetylene reducing activity of *A. evenia* plants inoculated with *Bradyrhizobium* ORS285, Δ*nifV* and Δ*nifV*+*nifV* derivatives at 22 dpi. The mean amount of produced ethylene per hour and per plant is indicated. Error bars represent standard errors of the mean (n = 10). An ANOVA was performed among the different conditions, followed by Tukey’s post hoc analysis (P < 0.05). The different letters above the bars indicate groups that differ significantly.

Dependent on the host plant, *Bradyrhizobium* ORS285 uses a NF-dependent or a NF-independent mechanism to establish a symbiotic interaction^10^. To investigate whether the absence of *nifV* affects the NF-independent interaction, we infected *A. evenia* with both the WT and the Δ*nifV* mutant. Observations done at 22 days post infection (dpi) showed drastic difference between the plants, the Δ*nifV* infected plants had typical nitrogen starvation symptoms such as foliar chlorosis and reduced plant growth as observed for the non-inoculated plant (Fig. 2A). Furthermore, while the plants infected with the WT strain harbor only red/pink colored nodules, nodules elicited by the Δ*nifV* mutant were heterogeneous in color and pink, yellow and green nodules were observed (Fig. 2A and B). The green color is indicative for leghaemoglobin degradation. Analyzing the kinetics of nodule formation showed that after 10 dpi the number of nodules on plants infected by the *ΔnifV* strain continued to increase whereas in plants infected by the WT strain the number of nodules started to stabilize. As a result, at 22 dpi, the number of nodules on Δ*nifV* infected plants was approximately twice the number found on plants inoculated with the WT strain (Fig. 2C). This increase in the nodule number is similar to what has been observed upon infection with nitrogenase minus mutants^11^, and could be attributed to a phenomenon termed autoregulation of nodulation that ensures a balance between nodule formation and energy requirements in legumes^12^. The acetylene reduction assay (ARA) showed that nodules of plants inoculated with the Δ*nifV* mutant strain had a very low nitrogenase enzyme activity (~10% of WT nodules; Fig. 2D) and produced “ethane” (Fig. S3A). All the observed phenotypes were absent when *A. evenia* plants were infected with an ORS285 Δ*nifV* strain that contained a plasmid carrying the *nifV* gene (Fig. 2A–D). Together, these data indicate that the *nifV* gene plays a critical role in the nitrogenase activity of the ORS285 strain during the symbiosis with *A. evenia*. The *Aeschynomene* species that form a NF-independent symbiotic interaction fall into a single clade^13^. To investigate whether the dependence on the rhizobial *nifV* gene is a generality within this clade, we infected nine other NF-independent species with the ORS285 Δ*nifV* mutant strain. With all tested species, plants inoculated with the Δ*nifV* mutant strain showed nitrogen starvation symptoms (Table 1). We must remark that for 3 species (*A. virginica*, *A. pratensis*, *A. selloi*) there was not a significant difference in the nitrogenase enzyme activity as measured by the ARA assay between plants inoculated with the WT and Δ*nifV* mutant strain. However, the formation of “ethane” in the ARA assay, an increased number of nodules, the presence of nodules with signs of senescence and the reduced stimulation of plant growth evidenced a nitrogenase enzyme complex that reduces dinitrogen inefficiently (Table 1). Taken together, these data indicate that the presence of the rhizobial homocitrate synthase NifV is required for an efficient symbiotic interaction with all tested NF-independent *Aeschynomene* species.

**Table 1:** Characteristics of the symbiotic interaction between different NF-independent *Aeschynomene* species and the WT and Δ*nifV* mutant of *Bradyrhizobium* ORS285.

Plant species	Growth stimulation plants		Mean number of nodules/plant		Legheamoglobin degradation (green nodules)		Nitrogenase enzyme activity (nmol ethylene/hr/plant)		“Ethane formation”	
	WT	Δ*nifV*	WT	Δ*nifV*	WT	Δ*nifV*	WT	Δ*nifV*	WT	Δ*nifV*
*A. evenia*	+++	−	17 ± 1 (A)	28 ± 1 (B)	no	yes	370 ± 28 (A)	27 ± 7 (B)	no	yes
*A. indica*	+++	−	33 ± 2 (A)	49 ± 3 (B)	no	yes	590 ± 26 (A)	340 ± 120 (B)	no	yes
*A. scabra*	+++	−	17 ± 1 (A)	33 ± 1 (B)	no	yes	534 ± 89 (A)	190 ± 17 (B)	no	yes
*A. sensitiva*	+++	−	14 ± 3 (A)	27 ± 3 (B)	no	yes	332 ± 34 (A)	69 ± 24 (B)	no	yes
*A. deamii*	+++	−	24 ± 1 (A)	45 ± 2 (B)	no	yes	665 ± 59 (A)	31 ± 10 (B)	no	yes
*A. denticulata*	+++	−	18 ± 1 (A)	48 ± 2 (B)	no	yes	402 ± 29 (A)	227 ± 30 (B)	no	yes
*A. virginica*	+++	+	39 ± 2 (A)	82 ± 3(B)	no	yes	867 ± 89 (A)	819 ± 35 (A)	no	yes
*A. tambacoudensis*	++	−	25 ± 3 (A)	49 ± 1(B)	no	yes	247 ± 13 (A)	80 ± 13 (B)	no	yes
*A. pratensis*	+++	+	11 ± 1 (A)	23 ± 1 (B)	no	yes	202 ± 62 (A)	151 ± 12 (A)	no	yes
*A. selloi*	+++	+	20 ± 2 (A)	29 ± 1 (B)	no	yes	574 ± 55 (A)	482 ± 34 (A)	no	yes

Seeds of different *Aeschynomene* species were sterilized, germinated and seedlings were inoculated with *Bradyrhizobium* ORS285 and *Bradyrhizobium* ORS285 Δ*nifV*, respectively. At 22 (*A. evenia*) or 28 dpi (others), the plant growth was compared with non–inoculated control plants and the mean nodule number, mature nodule phenotype and mean nitrogenase enzyme activity as analysed by the ARA assay was determined. +++: no N-starvation signs and plants are much better developed than the non-inoculated control plants; ++: no N-starvation signs and plants are better developed than the non-inoculated control plants; +: plants are better developed than the non-inoculated control plants but have signs of foliar chlorosis; −: no difference with non-inoculated control plants. ±Indicates the standard error of the mean. Letters in brackets in the table represent conditions with significant difference according to the Tukey’s test (P < 0.05).

### In NF-dependent *Aeschynomene* species the requirement for rhizobial NifV depends on the host plant

**Figure 3:**
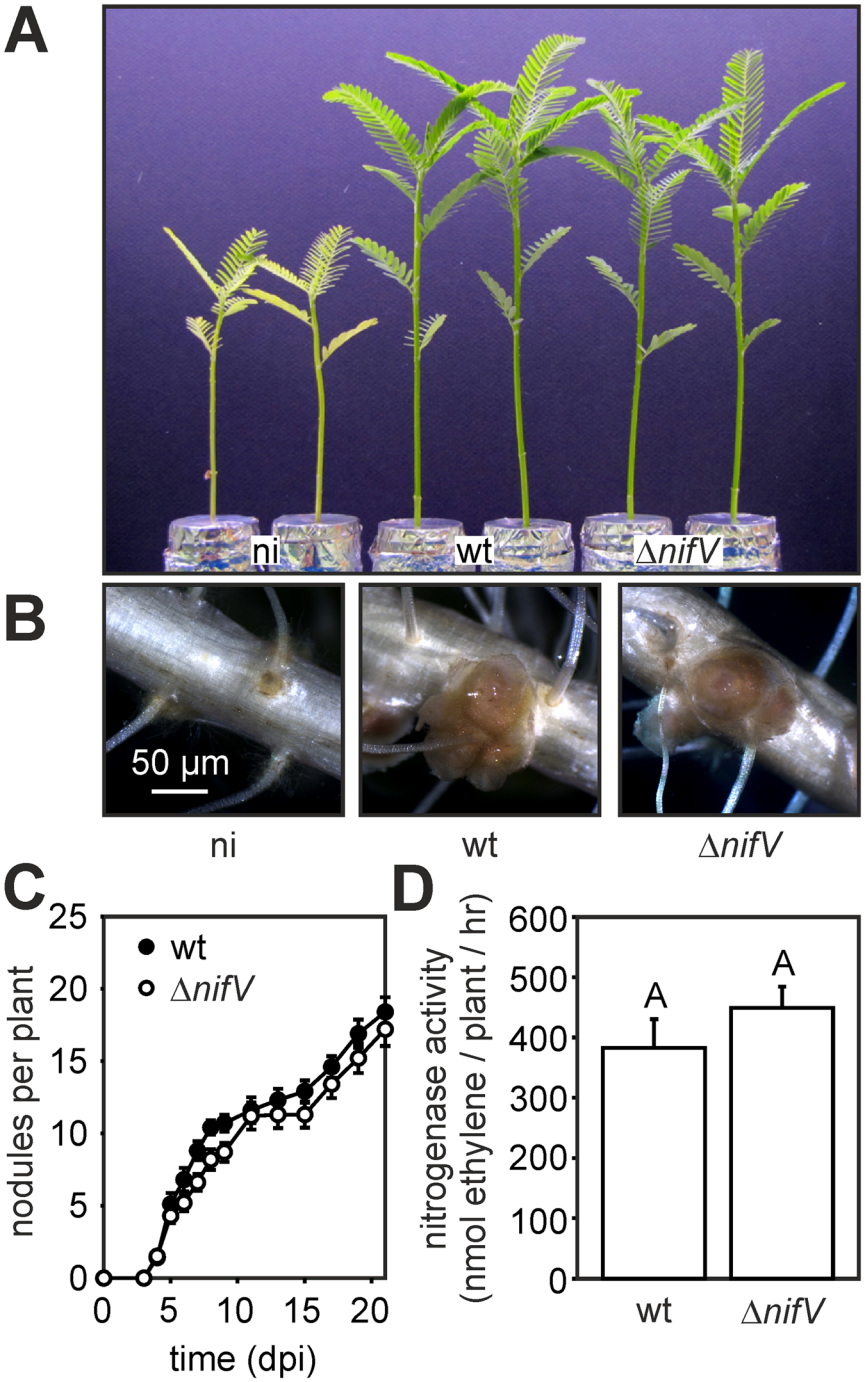
ORS285 *nifV* deletion does not affect the symbiotic interaction with *Aeschynomene afraspera* (LSTM #1). (**A**) Comparison of the growth of *A. afraspera* plants inoculated with ORS285 (wt) and ORS285 Δ*nifV*. Non-inoculated plants (ni) were used as control. (**B**) Mature nodules on *A. afraspera* plants inoculated with ORS285 and ORS285 Δ*nifV*. (**C**) Nodulation kinetics of *Bradyrhizobium* ORS285 (wt) and ORS285 Δ*nifV* (*∆nifV*) on *A. afraspera* plants. The mean number of nodules per plant (n = 10) at various days post infection (dpi) is presented. (**D**) Acetylene reducing activity of *A. afraspera* plants inoculated with *Bradyrhizobium* ORS285 (wt) and ORS285 Δ*nifV* at 21 dpi. The mean amount of produced ethylene per hour and per plant is indicated. Error bars represent standard errors of the mean (n = 10). Tukey’s post hoc analysis (P < 0.05) showed no significant difference in acetylene reduction between plants inoculated with the two strains.

**Figure 4:**
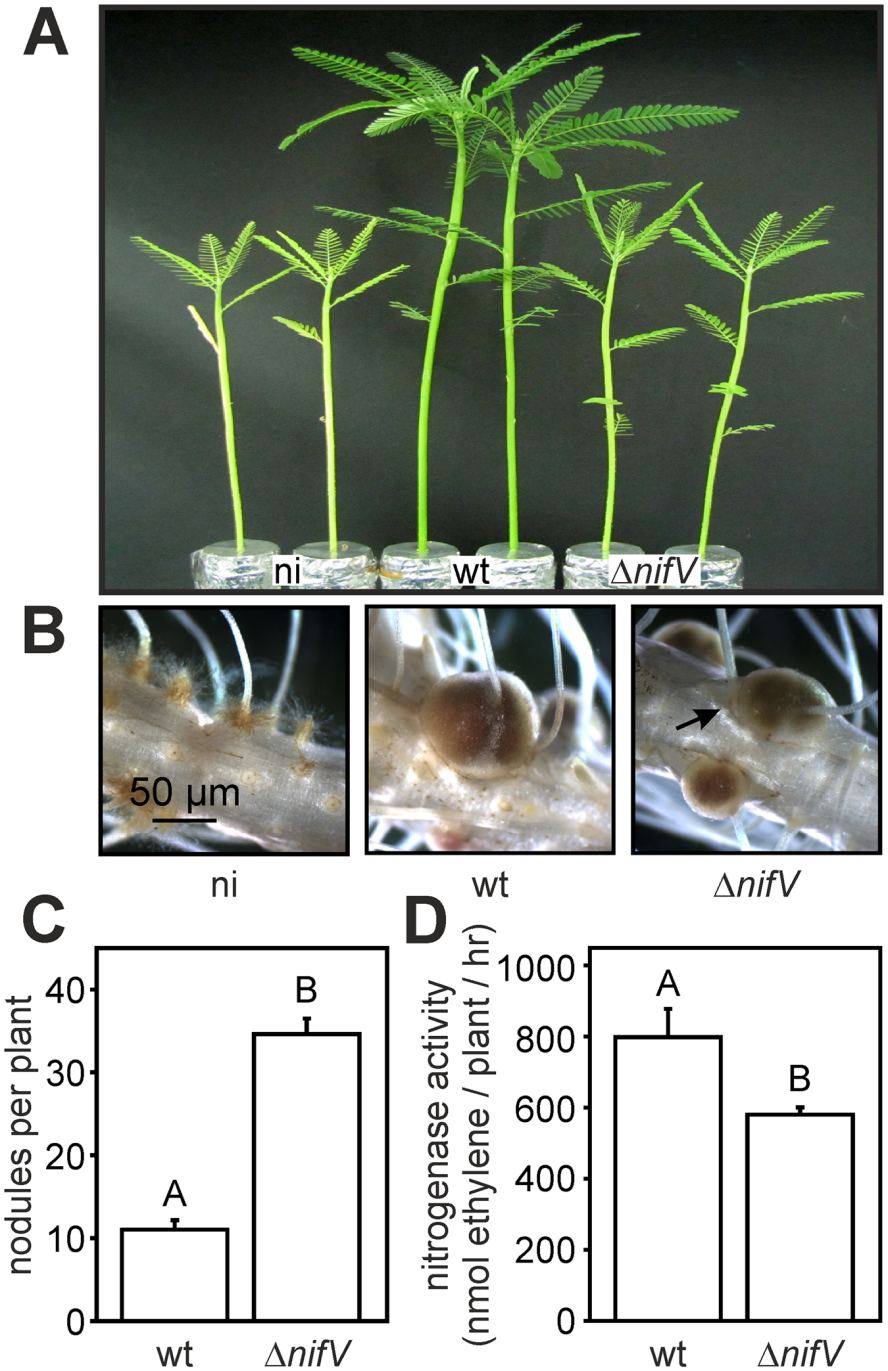
ORS285 *nifV* deletion affects the symbiotic interaction with *Aeschynomene nilotica* (IRRI 014040). (**A**) Comparison of the growth of *A. nilotica* plants inoculated with ORS285 and ORS285 Δ*nifV* at 28 days post infection (dpi). Non-inoculated plants (ni) were used as control. (**B**) Mature nodules on *A. nilotica* plants inoculated with ORS285 and ORS285 Δ*nifV*. Note the presence of green colored nodules (black arrow) on *A. nilotica* plants inoculated with the ORS285 Δ*nifV* strain. (**C**) Number of root nodules on *A. nilotica* plants inoculated with *Bradyrhizobium* ORS285 and *Bradyrhizobium* ORS285 Δ*nifV*, respectively. The mean number of nodules per plant (n = 5) at 28 dpi is presented. (**D**) Acetylene reducing activity of *A. nilotica* plants inoculated with *Bradyrhizobium* ORS285 and ORS285 Δ*nifV* at 28 dpi. The mean amount of produced ethylene per hour and per plant (n = 5) is indicated. In (C) and (D) error bars represent standard errors of the mean and letters represent conditions with significant difference according to the Tukey’s test (P < 0.05).

To investigate the role of NifV in the NF-dependent symbiotic interaction, we infected *A. afraspera* plant*s* with the ORS285 Δ*nifV* mutant. At 22 dpi, plants inoculated with the Δ*nifV* mutant were in all phenotypic aspects (growth, nodule number and color) indistinguishable from plants inoculated with the WT strain (Fig. 3A,B and C). Also no “ethane” formation was detected in the ARA assay (Fig. S3B) and the kinetics of nodule formation and nitrogenase activity were similar as observed with the WT strain (Fig. 3C and D). In the group of NF-dependent *Aeschynomene* species, only few can form efficient nitrogen fixing nodules with the ORS285 strain^13^. *A. nilotica* plants that belong to this small group, have typical nitrogen starvation symptoms and all other phenotypes as observed with the group of NF-independent *Aeschynomene* species when inoculated with the Δ*nifV* mutant (Fig. 4A–D,B; Fig. S3C). This indicates that in contrast to *A. afraspera*, *A. nilotica* plants are unable to compensate for the absence of NifV in ORS285. Thus, in the group of NF-dependent *Aeschynomene* species, the requirement of NifV for a symbiotic nitrogenase activity differs according to the host plant.

### *Aeschynomene evenia* and *Aeschynomene afraspera* contain putative orthologs of *Lotus japonicus* FEN1

**Figure 5:**
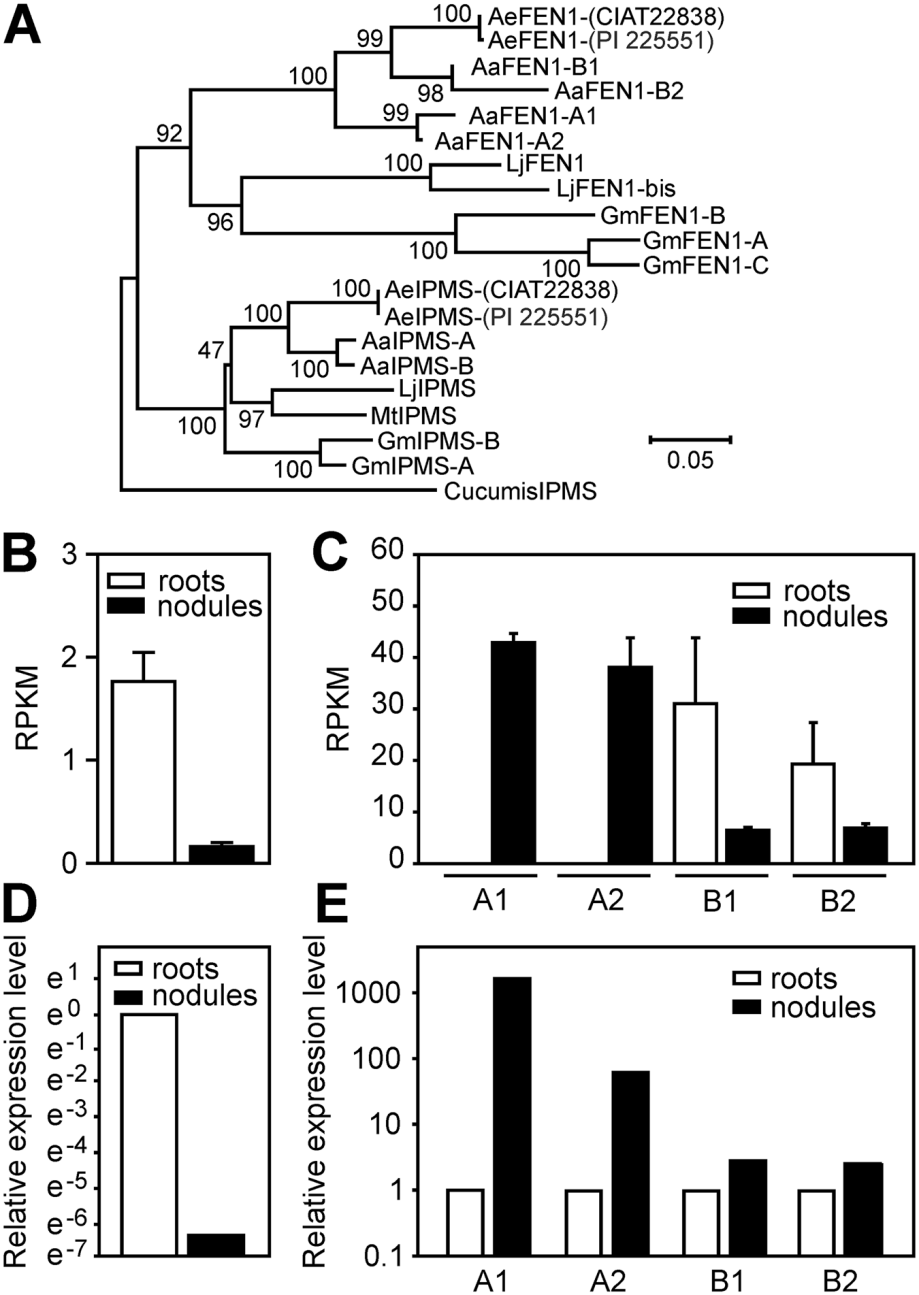
The *Aeschynomene* legumes *A. evenia* and *A. afraspera* contain *FEN1* homoloques but only nodule specific expression is observed in *A. afraspera* plants. (**A**) Phylogenie based on FEN1 and isopropylmalate synthase (IPMS) sequences obtained from genomic and transcriptome databases of *A. evenia* (CIAT22838/PI 225551) and *A. afraspera* (LSTM #1). *Cuccumis*, *Glycine max*, *Lotus japonicus* and *Medicago truncatula* IPMS and FEN1 sequences were obtained from Genebank. -A, -A1, -B, -B1, bis and -C indicate different copies found in (polyploid) species. Cuccumis IPMS was used as outgroup. Numbers at nodes represent bootstrap values (% of 1000 replicates). (**B**) Transcript abundance reads (reads per kilobase per million; RPKM) of *FEN1* in root and nodule tissue of *A. evenia* (PI 225551). (**C**) Transcript abundance reads (RPKM) of the different *FEN1* homologues in root and nodule tissue of *A. afraspera* (LSTM #1) Error bars as shown in (B) and (C) indicate standard errors of the means of three biological replicates. (**D**) Relative *FEN1* expression level in *A. evenia* (PI 225551) nodules elicited by *Bradyrhizobium* ORS285 at 8 dpi. (**E**) Relative expression level of the different *FEN1* homoloques in *A. afraspera* (LSTM #1) nodules elicited by *Bradyrhizobium* ORS285 at 8 dpi. The relative expression level of the different *FEN1* homologues as shown in (D), (E) was determined by RT-qPCR and normalized by the expression of Elongation Factor 1α. Non-inoculated roots were used as control.

*L. japonicus* and *G. max* express a nodule-specific homocitrate synthase gene annotated respectively FEN1 and GmN56^5, 14^. Interestingly, previous analyses of the transcriptomes of *A. evenia* (CIAT22838) that included nodule tissue failed to identify a *FEN1* ortholog^15^. In order to analyze the presence and expression of *FEN1*-like genes in different *Aeschynomene* species, we made use of genomic data obtained in the frame of an ongoing genome sequencing project for *A. evenia* (CIAT22838) to perform a blast search using *L. japonicus* FEN1 as query. This resulted in one gene-product that after phylogenetic analysis clustered in a clade with FEN1 of *L. japonicus* (Fig. 5A). By PCR, cloning, sequencing and transriptomic data analysis (see material and method section SI) using this *A. evenia* (CIAT22838) *FEN1* sequence as basis, we obtained four different sequences of putative *FEN1* genes for *A. afraspera* and one sequence for another *A. evenia* line, PI 225551. This difference in the number of FEN1 homologs between these two species is not surprising considering that *A. evenia* is diploid whereas *A. afraspera* is octoploid^16^. Phylogenetic analysis showed that all the gene products clustered in a clade containing *L. japonicus* FEN1 (Fig. 5A). This suggests that *FEN1* is present in a single copy in the 2x *A. evenia* genome and that 4 paralogs are present in the 8x *A. afraspera* genome.

Analyzing transcriptomic data showed that in *A. evenia* (PI 225551) the expression level of the identified *FEN1* gene is low and not nodule-specific (Fig. 5B), in accordance with the reported absence of expression for the other *A. evenia* line CIAT22838^15^. However, in *A. afraspera* two of the four *FEN1* copies (A1 and A2) are highly expressed specifically in nodules during symbiosis with *Bradyrhizobium* ORS285 (Fig. 5C). RT-Q-PCR analysis using specific primers for the individual *FEN1* copies confirmed the experimental data as obtained by RNA-seq analysis (Fig. 5D and E). Hence, for these two tested *Aeschynomene* species, the absence of *FEN1* expression correlates with the NifV requirement for efficient nitrogen fixation in nodules.

## Discussion

How do rhizobia fix nitrogen during symbiosis while they lack the *nifV* gene that is required for homocitrate synthesis, an essential co-factor of the nitrogenase enzyme complex? This important issue has been resolved in the case of the *L. japonicus* – *Mesorhizobium loti* symbiosis for which it has been shown that the plant overcomes the lack of *nifV* in the bacterial partner via a nodule specific expression of an homocitrate synthase homolog, FEN1^5^. However, the question remains if this plant homocitrate supplementation constitutes a general paradigm for all the rhizobium/legume symbioses. This question is particularly meaningful in the case of symbiotic interactions involving rhizobia that do contain a *nifV* gene, such as photosynthetic bradyrhizobia and *A. caulinodans*.

Here, by studying the symbiotic interaction of a *nifV* mutant of the photosynthetic *Bradyrhizobium* strain ORS285 and different *Aeschynomene* species, we demonstrate that not all legumeous plants supply the rhizobial partner with (sufficient) homocitrate for nitrogen fixation. In particular for all the NF-independent *Aeschynomene* species tested, we observed an important effect of the Δ*nifV* mutation on the symbiotic efficiency indicating the inability of these plant species to compensate for the absence of *nifV* (Fig. 2 and Table 1). It is to note that for 3 out of the 9 species tested, the symptoms of the Δ*nifV* mutation are not as drastic as observed for *A. evenia* (Table 1). In these cases the measured nitrogenase enzyme activity of the Δ*nifV* inoculated plants was close to these of the WT strain and a weak benefit on the plant growth by inoculation with the Δ*nifV* mutant was observed. The nitrogenase enzyme activity as measured in the ARA assay, suggest that the nitrogenase enzyme in Δ*nifV* induced nodules of these species is fully functional. However, the readily detected and proportionally increased amounts of “ethane” indicate that the nitrogenase enzyme complex (FeMo-cofactor) in nodules formed by Δ*nifV* mutant is different from the one in nodules induced by the WT strain. Moreover, the number of nodules formed by the Δ*nifV* mutant is double the amount formed by the WT strain and some of these Δ*nifV* induced nodules show signs of senescene. Thus, in contrast to the measured nitrogenase activity, all other observations with these three *Aeschynomene* species indicate that the symbiotic nitrogen fixation of the Δ*nifV* mutant is very inefficient. This difference can be explained by the fact that the nitrogenase activity as measured in the ARA assay (=reduction of only one of the triple bonds in acetylene) is not directly related to symbiotic nitrogen fixation which requires reduction of all triple bonds in dinitrogen. As shown in Fig. 1, in free-living conditions the nitrogenase activity of the Δ*nifV* mutant is not zero. Thus, when plants do not (or less rapid) sanction nodules that fix dinitrogen inefficiently, an increase in nodule number can give raise to a nitrogenase activity in the ARA assay which is close the one as observed for WT nodules. We hypothesize that the latter is the case in the three NF-independent *Aeschynomene* species showing a high nitrogenase activity with the Δ*nifV* mutant.

Fascinatingly, when the Δ*nifV* mutant is tested on the NF-dependent *Aeschynomene* species, *A. afraspera*, no effect of the mutation is detected indicating that the plant overcomes the absence of *nifV* in contrast to what is observed for *A. evenia* (Fig. 3). This difference between the two species is directly correlated with *FEN1* expression. While two of the four *FEN1* orthologs (*FEN1*-A1/A2) identified in *A. afraspera* genome (8x) are specifically expressed in the nodules (Fig. 5C and F), the only *FEN1* ortholog identified in *A. evenia* (2x) displayed an opposite pattern of expression (down-expression in nodules) (Fig. 5B and D).

This non-requirement of bacterial NifV for symbiotic dinitrogen fixation is not a general feature of NF-dependent *Aeschynomene* species because in the second species tested, *A. nilotica*, we observed a drastic effect of the Δ*nifV* mutation on symbiotic efficiency (Fig. 4). The contrasting observations within the NF-dependent group render it difficult to propose a simple evolutionary scenario to explain the observed differences between *Aeschynomene* species. Nevertheless, knowledge acquired on the diversity and ecology of the *Aeschynomene/Bradyrhizobium* symbiosis could give some hints. It has been proposed that FEN1 was recruited from a housekeeping gene encoding an IPMS during the evolution of symbiosis^5^. The acquisition of this property, the specific control of *FEN1* expression in the nodules, and a subsequent loss of *nifV* in the symbiont gives the host plant the capacity to control where and when the rhizobia make an active nitrogenase. Considering the high energy demand of the nitrogenase, this control represents a clear functional advantage. In nature, the NF-independent *Aeschynomene* species interact specifically with photosynthetic bradyrhizobia and these bacteria are known to nodulate only this group of plants. In addition, unlike other legumes, this group of *Aeschynomene* species form stem nodules. There are two possible explanations why the *nifV* genes are maintained in these bacteria. First, the photosynthetic properties of these bradyrhizobial strains make that in case of stem nodules they are less dependent on energy furnished by the plant for nitrogen fixation^10^. The need for the plant to control the energy consumption by the nodule tissue via homocitrate synthesis is thus less significant. Second, the ability of the bacteria to nodulate the stem, a surrounding very poor in nutrients makes the capacity to fix dinitrogen under free-living conditions an advantage for the bacterium as it will increase survival and infectivity. In the same vein, it is to highlight that *A.caulinodans* that also contains a *nifV* gene forms stems nodules on *Sesbania rostrata*.

In the group of NF-dependent *Aeschynomene* species, only a few members form stem nodules. In addition, the bacterial partner choice is less specific and symbiotic interactions with both photosynthetic and non-photosynthetic *Bradyrhizobium* strains are possible. Some of these non-photosynthetic strains (for example *Bradyrhizobium* USDA110^17^) do not contain a *nifV* gene. As NF-dependent *Aeschynomene* species show more heterogeneous symbiotic traits, the ability to overcome the lack of *nifV* in their bacterial partner may have a selective advantage.

Here, we show that in contrast to the model legume *L. japonicus* many *Aeschynomene* species do not supply homocitrate to the rhizobial partner during symbiosis. The subsequent question that arises is: Is *Aeschynomene*/photosynthetic *Bradyrhizobium* symbiosis an atypical example or are there other rhizobia/legume symbiosises that require rhizobial homocitrate synthesis to be efficient? As indicated above, the *A. caulinodans/Sesbania rostrata* symbiosis might be a second example, but what else? A survey of the genome sequences available show that ±10% of the sequenced rhizobia do contain a gene that is annotated as homocitrate synthase (Table S1). It would be very interesting to analyze if these rhizobia like photosynthetic bradyrhizobia are able to fix dinitrogen under free-living conditions and/or if the presence of the *nifV* gene is related to the absence of *FEN1* expression by their natural host plant(s) like we observed for *A. evenia*. This knowledge will deepen our understanding on the evolution of the rhizobium / legume symbiosis and could contribute to a better selection of nitrogen fixing inoculum strains.

## Methods

### Bacterial strains and growth conditions

A detailed description of the construction of a *nifV* deletion strain can be found in the supplementary information section. *Bradyrhizobium* ORS285 and derivatives were grown in modified YM medium^18^ or BNM-B medium^19^. *Escherichia coli* strains were grown in Luria-Bertani medium (LB) at 37 °C. When required, the media were supplemented with kanamycin (100 μg/ml) or a mixture of kanamycin (120 μg/ml) and cefotaxime (20 μg/ml) for the selection of ORS285 clones in conjugation experiments.

### Plant growth and acetylene reduction assay

A table of *Aeschynomene* species used in this study and their origin can be found in the Supplementary information section (Table S2). Sterilization of seeds, germination, plant growth and inoculation with bacterial strains were as described^17^. At the indicated times after inoculation as specified in the figure legends, photos of plants were taken, the number of nodules on the roots were counted and the acetylene reduction assay (ARA) was used to measure the nitrogenase enzyme activity^17^.

### *In vitro* nitrogenase enzyme activity

Bacterial cultures were grown in liquid BNM-B medium containing 10 mM succinate under anoxic conditions and 10% acetylene. At the indicated times the amount of ethylene produced by the bacterial culture was measured by gas chromatography^17^. For complementation studies, homocitrate (Sigma-Aldrich; 10 mM final concentration) was added to the growth medium.

### Transcriptome and real-time quantitative PCR expression analysis

Transcriptomic data for nodules of *A. evenia* (PI 225551) and *A. afraspera* (LSTM #1) were obtained and analysed as decribed^15^. Total RNA was extracted from lateral root regions (non-inoculated plant; ±1 cm around the exit of lateral roots) or nodules (plants inoculated with *Bradyrhizobium* ORS285) at 8 days after inoculation using the SV Total RNA Isolation system (Promega). Quantification of RNA, reverse transcription, real-time quantitative PCR and analysis of the data was performed as describe before^20^. Primers for quantitative PCR can be found in Table S3 the Supplementary information section.

### Phylogenetic analysis

The procedure for the identification of *FEN1* and IPMS orthologs in *A. evenia* (CIAT22838/PI 225551) and *A. afraspera* ((LSTM #1) is described in detail in the Supplementary information section. Using the obtained sequences a phylogenetic analysis was performed as described in^16^ and data are presented as rooted trees using the *Cucumis* IPMS as outgroup. GenBank/EMBL and Gene_ID numbers for sequences obtained and used for phylogenetic analysis can be found in Table S4 of the Supplementary information section. All DNA sequences generated in this study are deposited in Genbank under accession numbers KY412790–KY412799 and KY618805–KY618808.

## Electronic supplementary material

Supplementary Information

## References

[1] Cheng Q (2008). Perspectives in biological nitrogen fixation research. J. Integr. Plant Biol..

[2] Rubio LM, Ludden PW (2008). Biosynthesis of the iron-molybdenum cofactor of nitrogenase. Annu. Rev. Microbiol..

[3] Masson-Boivin C, Giraud E, Perret X, Batut J (2009). Establishing nitrogen-fixing symbiosis with legumes: how many rhizobium recipes?. Trends Microbiol..

[4] Hoover TR, Imperial J, Ludden PW, Shah VK (1989). Homocitrate is a component of the iron-molybdenum cofactor of nitrogenase. Biochemistry.

[5] Hakoyama T (2009). Host plant genome overcomes the lack of a bacterial gene for symbiotic nitrogen fixation. Nature.

[6] Dreyfus BL, Elmerich C, Dommergues YR (1983). Free-living *Rhizobium* strain able to grow on N(2) as the sole nitrogen source. Appl. Environ. Microbiol..

[7] Alazard D (1990). Nitrogen fixation in pure culture by rhizobia isolated from stem nodules of tropical *Aeschynomene* species. FEMS Microbiol. Lett.

[8] Hoover TR, Imperial J, Ludden PW, Shah VK (1988). Homocitrate cures the NifV- phenotype in *Klebsiella pneumoniae*. J. Bacteriol..

[9] Scott DJ, Dean DR, Newton WE (1992). Nitrogenase-catalyzed ethane production and CO-sensitive hydrogen evolution from MoFe proteins having amino acid substitutions in an alpha-subunit FeMo cofactor-binding domain. J. Biol. Chem..

[10] Giraud E (2007). Legumes symbioses: absence of *nod* genes in photosynthetic bradyrhizobia. Science.

[11] Gourion B (2011). Bacterial RuBisCO is required for efficient *Bradyrhizobium*/*Aeschynomene* symbiosis. PLoS One.

[12] Mortier V, Holsters M, Goormachtig S (2012). Never too many? How legumes control nodule numbers. Plant. Cell Environ..

[13] Chaintreuil C (2013). Evolution of symbiosis in the legume genus. Aeschynomene. New Phytol..

[14] Kouchi H, Hata S (1995). GmN56, a novel nodule-specific cDNA from soybean root nodules encodes a protein homologous to isopropylmalate synthase and homocitrate synthase. Mol. Plant. Microbe. Interact..

[15] Chaintreuil C (2016). A gene-based map of the Nod factor-independent *Aeschynomene evenia* sheds new light on the evolution of nodulation and legume genomes. DNA Res..

[16] Chaintreuil C (2016). The evolutionary dynamics of ancient and recent polyploidy in the African semiaquatic species of the legume genus. Aeschynomene. New Phytol..

[17] Giraud E, Hannibal L, Fardoux J, Verméglio A, Dreyfus B (2000). Effect of *Bradyrhizobium* photosynthesis on stem nodulation of *Aeschynomene sensitiva*. Proc. Natl. Acad. Sci. USA.

[18] Nouwen N, Fardoux J, Giraud E (2016). NodD1 and NodD2 are not required for the symbiotic interaction of *Bradyrhizobium* ORS285 with Nod-factor-independent *Aeschynomene* legumes. PLoS One.

[19] Renier A (2011). Photosynthetic *Bradyrhizobium* sp. strain ORS285 synthesizes 2-O-methylfucosylated lipochitooligosaccharides for *nod* gene-dependent interaction with *Aeschynomene* plants. Mol. Plant. Microbe. Interact..

[20] Czernic P (2015). Convergent evolution of endosymbiont differentiation in Dalbergioid and Inverted Repeat-Lacking Clade legumes mediated by nodule-specific cysteine-rich peptides. Plant Physiol..

