# Supplementary material for: The role of rhizobial (NifV) and plant (FEN1) homocitrate synthases in Aeschynomene/photosynthetic Bradyrhizobium symbiosis
Source: Sci Rep. 2017 Mar 27;7:448. doi: 10.1038/s41598-017-00559-0 (PMC5428708; doi:10.1038/s41598-017-00559-0)
Supplement: Supplementary file 1 — Supplementary Information [file 41598_2017_559_MOESM1_ESM.pdf]

The role of rhizobial (NifV) and plant (FEN1) homocitrate synthases in *Aeschynomene* / photosynthetic *Bradyrhizobium* symbiosis.

Nico Nouwen, Jean-François Arrighi, Fabienne Cartieaux, Clémence Chaintreuil, Djamel Gully, Christophe Klopp and Eric Giraud

Supplementary information

Methods

Bacterial strains.

To construct a deletion of the *nifV* gene of *Bradyrhizobium* ORS285 by crossover PCR the following sets of primers were used. fur-*Xho*I: 5' CTCGAGTCTCGCAGGCCACCATCTAC-3'; nifV-del-REV: 5'-CTTCGCGCCAGATGGATCCGAGCGTGGTGTCGTTCAGGATG-3'; nifV-del-FOR: 5'-GACACCACGCTCGGATCCATCTGGCGCGAAGTCTGTGAT-3' and cysE-rev: 5'-TTGGATGACGACCGAATTGG-3'. The resulting DNA fragment was cloned into pGEM®-T Easy (Promega) and transformed into thermocompetent *E.coli* XL2-Blue (Agilent Genomics) cells. Correct clones were verified by sequence analysis. For *nifV* deletion, the Δ*nifV* fragment in pGEM®-T Easy was excised with *Apa*I - *Spe*I and ligated into the suicide vector pNPTS139 [1] digested with *Apa*I – *Spe*I. The ligation mixture was transformed into *E.coli* XL2-Blue cells and correct clones were selected via kanamycin resistance (100 µg/ml; pNPTS139-Δ*nifV*) and subsequent DNA restriction enzyme analysis. For mobilization into *Bradyrhizobium* ORS285 [2], plasmid pNPTS139-Δ*nifV*, which contains a counter selectable *sacB* marker, was transformed into $CaCl_2$ competent *E.coli* S17.1 cells. Conjugation, was performed using the biparental mating protocol as previously described [3]. The *nifV* deletion in sucrose resistant and kanamycin sensitive colonies was confirmed by PCR.

***In vitro* nitrogenase enzyme activity.**

Bacterial cultures grown in YM medium were washed with BNM medium and subsequently suspended in BNM-B medium containing 10 mM succinate (start optical density at 600 nm: ~ 0.12). This culture was used to inoculate 150 ml glass vials sealed with rubber septa (50 ml inoculum) or 9 ml vacuette® tubes (Greiner bio-one; ref:455001; 1 ml inoculum). To avoid overpressure, 15 ml / 0.9 ml of air was removed before injecting the same volume of 100% acetylene. The cultures were incubated at 28°C without shaking. In kinetic studies, at different times the absorbance (at 600 nm) of the bacterial culture was measured by taking aseptically a 1 ml sample. In addition, the amount of ethylene produced by the bacterial culture was measured by injecting 1 ml gas sample into a gas chromatograph. For complementation studies, homocitrate (Sigma-Aldrich; 10 mM final concentration) was added to the growth medium.

Molecular methods and sequence analysis.

*FEN*1 was identified in the *A. evenia* (CIAT22838) genome (J. F. Arrighi, unpublished) by Blast search using the FEN1 sequence of *L. japonicus* as query. Using the *A. evenia FEN1* genomic sequence, primers were designed to amplify the first exon of all *FEN1* orthologs in *A. afraspera*. FEN1-F3 : 5'-CCCACACTACATTCCCAACC-3' and FEN1-R1 : 5'-GCAGCATCTTCAGGGACAA-3' (A1; A2) and primers FEN1-F3 : 5'-CCCACACTACATTCCCAACC-3'and FEN1-R2 : 5'-ATCATCGCAACCAAGACTCC-3' (B1; B2). PCR amplifications, cloning and sequencing of PCR products were performed as described in [45]. The generated DNA sequences are deposited in Genbank under accession numbers KY412792 – KY412794. To identify the complete coding sequence of *A. afraspera* (LSTM #1) and *A. evenia var. evenia* (PI 225551) *FEN1* orthologs, the sequence obtained for

the first exon and *A. evenia* (CIAT22838) *FEN*1 genomic sequence, respectively, were used as query in a BLAST search in *A. afraspera* and *A. evenia var evenia* transcriptomes ([6]; unpublished results). To identify in the transcriptomes genes encoding isopropylmalate synthases (IPMS), the sequence of *L. japonicus* IPMS (Lj5g3v2298240) was used as query in a BLAST search.

References

1. Fischer, B., Rummel, G., Aldridge, P. & Jenal, U. The FtsH protease is involved in development, stress response and heat shock control in *Caulobacter crescentus*. *Mol. Microbiol.* **44,** 461–478 (2002).
2. Molouba, F. *et al.* Photosynthetic bradyrhizobia from *Aeschynomene* spp. are specific to stem-nodulated species and form a separate 16S ribosomal DNA restriction fragment length polymorphism group. *Appl. Environ. Microbiol.* **65,** 3084–94 (1999).
3. Podlešáková, K. *et al.* Rhizobial synthesized cytokinins contribute to but are not essential for the symbiotic interaction between photosynthetic bradyrhizobia and *Aeschynomene* legumes. *Mol. Plant-Microbe Interact.* **26,** 1232–1238 (2013).
4. Arrighi, J.-F. *et al.* Genotype delimitation in the Nod-independent model legume *Aeschynomene evenia*. *PLoS One* **8,** e63836 (2013).
5. Arrighi, J.-F. *et al.* Radiation of the Nod-independent *Aeschynomene* relies on multiple allopolyploid speciation events. *New Phytol.* **201,** 1457–1468 (2014).
6. Czernic, P. *et al.* Convergent evolution of endosymbiont differentiation in Dalbergioid and Inverted Repeat-Lacking Clade legumes mediated by nodule-specific cysteine-rich peptides. *Plant Physiol.* **169,** 1254–1265 (2015).

Table S1. Gene products annotated as homocitrate synthase in rhizobial genomes that are present in the genome portal of the U.S. Departement of Energy Joint Genome Institute (DOE JGI). The rhizobial genomic database was searched with homocitrate synthase as keyword. As homocitrate synthases belong to the same enzyme family as isopropyl malate synthases, strains in which the gene product was annotated as putative alpha-isopropylmalate / homocitrate synthase family protein were ommited from the table. In addition, for the rhizobial strains containing a gene product annotated as homocitrate synthase it was verified that they contained at least one other gene of which the product had been annotated as alpha-isopropylmalate (/homocitrate synthase) family protein.

**Genome**	**Locus Tag**	**Gene Product Name**
Azorhizobium caulinodans ORS571	AZC_3389	putative homocitrate synthase
Azorhizobium doebereinerae UFLA1-100	YU1DRAFT_01815	homocitrate synthase NifV
Bradyrhizobium elkanii 587	BELKANIIDRAFT_04289	homocitrate synthase NifV
Bradyrhizobium elkanii USDA76	BraelDRAFT_3014	homocitrate synthase NifV
Bradyrhizobium elkanii USDA 94	A3AKDRAFT_06922	homocitrate synthase NifV
Bradyrhizobium elkanii USDA 3259	YUGDRAFT_06316	homocitrate synthase NifV
Bradyrhizobium elkanii WSM2783	YY7DRAFT_09596	homocitrate synthase NifV
Bradyrhizobium elkanii WSM1741	YUODRAFT_06272	homocitrate synthase NifV
Bradyrhizobium elkanii USDA 3254	A3AMDRAFT_07009	homocitrate synthase NifV
Bradyrhizobium genosp. SA-4 CB756	BrageDRAFT_6259	homocitrate synthase NifV
Bradyrhizobium japonicum 22	K410DRAFT_4369	homocitrate synthase NifV
Bradyrhizobium sp. Ai1a-2	K288DRAFT_07984	homocitrate synthase NifV
Bradyrhizobium sp. ARR65	BraARR65DRAFT_00070590	homocitrate synthase NifV
Bradyrhizobium sp. BTAi1	BBta_5875	homocitrate synthase NifV
Bradyrhizobium sp. CCGE-LA001	BCCGELA001_11941	homocitrate synthase
Bradyrhizobium sp. Cp5.3	K289DRAFT_07402	homocitrate synthase NifV
Bradyrhizobium sp. DOA9	BDOA9DRAFT_06234	homocitrate synthase NifV
Bradyrhizobium sp. EC3.3	YUUDRAFT_06493	homocitrate synthase NifV
Bradyrhizobium sp. ORS278	BRADO5390	homocitrate synthase NifV
Bradyrhizobium sp. S23321	S23_45990	putative homocitrate synthase
Bradyrhizobium sp. TV2a.2	A3AIDRAFT_07842	homocitrate synthase NifV
Bradyrhizobium sp. USDA 3384	A3CKDRAFT_07072	homocitrate synthase NifV
Bradyrhizobium sp. WSM1743	YU9DRAFT_00569	homocitrate synthase NifV
Bradyrhizobium sp. WSM2254	A3M7DRAFT_05034	homocitrate synthase NifV
Bradyrhizobium sp. WSM2793	A3ASDRAFT_06087	homocitrate synthase NifV
Bradyrhizobium sp. WSM3983	YUADRAFT_06708	homocitrate synthase NifV
Bradyrhizobium sp. WSM3983	YUADRAFT_07328	homocitrate synthase NifV
Mesorhizobium amorphae CCNWGS0123	MEA186_26726	homocitrate synthase
Mesorhizobium sp. WSM3224	YU3DRAFT_05384	homocitrate synthase NifV
Rhizobium selenitireducens ATCC BAA-1503	L867DRAFT_04253	homocitrate synthase NifV

Table S2. Accessions and origin of *Aeschynomene* spp. used in this study.

***Aeschynomene* species**	**Accession**	**LSTM code**	**Origin**
**NF-dependent species**			
A. afraspera	LSTM #1	1	Senegal
A. nilotica	IRRI 014040	53	Senegal
**NF-independent species**			
A. evenia	CIAT22838	76	Malawi
A. evenia var. evenia	PI 225551	21	Zambia
A. indica	LSTM #19	19	Senegal
A. scabra	LSTM #26	26	Mexico
A. sensitiva	LSTM #28	28	Senegal
A. deamii	LSTM #24	24	Mexico
A. denticulata	IRRI 013003	50	Brazil
A. virginica	Essex Co. Virginica #42	63	USA
A. tambacoudensis	LSTM #60	60	Senegal
A. pratensis	IRRI 013006	55	Brazil
A. selloi	CPI 104040	186	Argentina

Table S3. List of primers used in this study for RT-qPCR experiments. The same primer pair designed for EF1α is used for *A. evenia* and *A. afraspera*.

**Gene**	**Forward primer**	**Reverse primer**
*EF*1α	AATGGTGATGCTGGTATGGTTAAG	TCTTCTTCTGTGCTGCCTTGG
*AeFEN1*_21	TTGGTTCAGGATCAGTGGATTCAG	TGGCAATCTCATCAACACCTTCT
*AaFEN1*_A1	ACTATGTCGGTGATGATGGCTATA	CCTCCTTCGTCTTCTTCAACTTG
*AaFEN1*_A2	GGAGCATAAGTTGAAGAAGACGAAA	ACAAACTGAACATCATCGCATCCC
*AaFEN1*_B1	AGAAGCTCGACATCGCACGC	AACAGGAACATAACCATCAACACC
*AaFEN1*_B2	AGGAGTCTTGGTTGCGATGATG	ATGCCAACAGTGTCAGGTATATCC

Table S4. Genbank number or Gene_ID for sequences used in the phylogenetic analysis.

**Species**	**accession:**	***FEN1***	***IPMS***
A. evenia	CIAT22838	KY412790	KY618805
	PI 225551	KY412797	KY618806
A. afraspera	LSTM1	A1: KY412795	A: KY618807
		A2: KY412798	B: KY618808
		B1: KY412799	
		B2: KY412796	
L. japonicus	MG-20	Lj1g3v3690020	Lj5g3v2298240
		bis: Lj1g3v3690180	
G. max	William 82	A: Glyma.19g120400	A: Glyma.20G245300
		B: Glyma.19g120600	B: Glyma.10G295400
		C: Glyma.03g005700	
M. truncatula	A17		Medtr1g116500
Cucumis	Gy14		LOC101205698

SI Figure legends

Fig. S1. *Bradyrhizobium* ORS285 *nifV* is localized in a genomic region containing other *nif* genes. Schematic overview of the genomic region surrounding the *nifV* gene. *irr*: putative iron response regulator

Fig. S2. Homocitrate addition to the culture medium or re-introducing *nifV* on a plasmid results in the loss of “ethane” production by ORS285 Δ*nifV* cells in the acetylene reduction assay. (A) chromatogram of gas-samples taken from ORS285 Δ*nifV* cells grown for 7 days in vacuette® tubes containing BNM-B medium and 10% acetylene gas in the absence (solid line) or presence (dashed line) of 10 mM homocitrate. (B) Chromatogram of gas-samples taken from ORS285 Δ*nifV* cells that do not (solid line) or do (dashed line) contain a plasmid containing *nifV* and which are grown for 7 days in vacuette® tubes containing BNM-B medium and 10% acetylene gas.

Fig. S3. *A. evenia* and *A. nilotica* plants that are inoculated with the *Bradyrhizobium* ORS285 Δ*nifV* strain produce “ethane” in the acetylene reduction assay. Chromatograms of gas-samples taken from (A) *A. evenia*, (B) *A. afraspera* and (C) *A. nilotica* plants inoculated with the WT (black line) or Δ*nifV* mutant (red line) at 22 / 28 dpi and incubated for 2 hours in the presence of 10% acetylene.

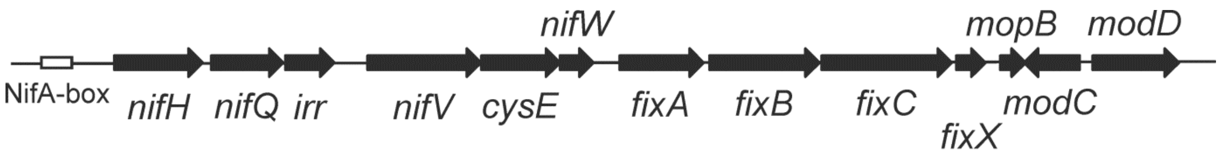
NifA-box
nifH
nifQ
irr
nifV
cysE
nifW
fixA
fixB
fixC
fixX
mopB
modC
modD

Figure S1

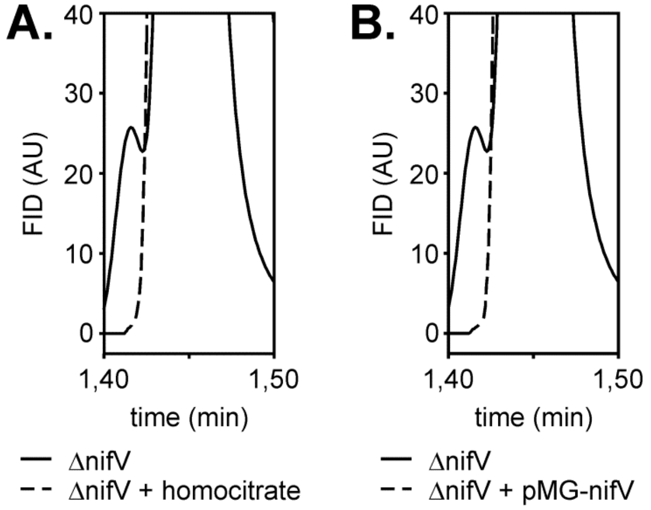
A.
B.
FID (AU)
40
30
20
10
0
1,40
1,50
time (min)
ΔnifV
ΔnifV + homocitrate
ΔnifV
ΔnifV + pMG-nifV

Figure S2

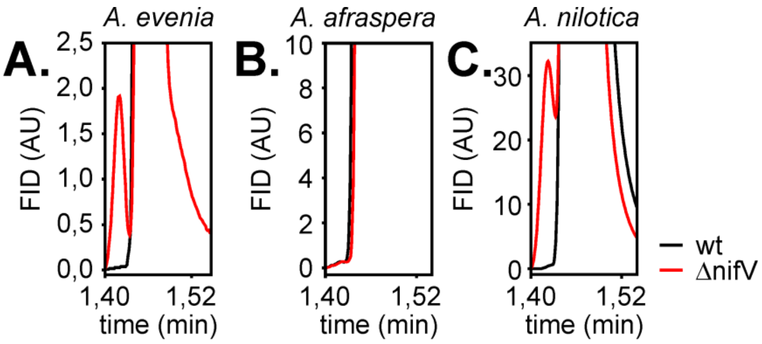
A. evenia
A.
2,5
2,0
1,5
1,0
0,5
0,0
FID (AU)
1,40
1,52
time (min)
A. afraspera
B.
10
8
6
4
2
0
FID (AU)
1,40
1,52
time (min)
A. nilotica
C.
30
20
10
0
FID (AU)
1,40
1,52
time (min)
wt
ΔnifV

Figure S3
